# Supplementary material for: High variability of dung beetle diversity patterns at four mountains of the Trans-Mexican Volcanic Belt
Source: PeerJ. 2018 Feb 27;6:e4468. doi: 10.7717/peerj.4468 (PMC5833475; doi:10.7717/peerj.4468)
Supplement: Supplemental Information 1 [file peerj-06-4468-s001.docx]

**Supplementary Material**

|   a | Tukey multiple comparisons of means 95% family-wise confidence level  Fit: aov(formula = distances ~ group, data = df)   \| $group \| diff \| lwr \| upr \| p adj \| \| --- \| --- \| --- \| --- \| --- \| \| CP-MA \| 0.10 \| -0.17 \| 0.37 \| 0.71 \| \| PO-MA \| 0.10 \| -0.17 \| 0.37 \| 0.67 \| \| SN-MA \| 0.12 \| -0.15 \| 0.39 \| 0.54 \| \| PO-CP \| 0.01 \| -0.26 \| 0.27 \| 1.00 \| \| SN-CP \| 0.03 \| -0.24 \| 0.29 \| 0.99 \| \| SN-PO \| 0.02 \| -0.25 \| 0.29 \| 1.00 \| | |
| --- | --- | --- | --- | --- | --- | --- | --- | --- | --- | --- | --- | --- | --- | --- | --- | --- | --- | --- | --- | --- | --- | --- | --- | --- | --- | --- | --- | --- | --- | --- | --- | --- | --- | --- | --- | --- | --- |
|   b | | \| $group \| diff \| lwr \| upr \| p adj \| \| --- \| --- \| --- \| --- \| --- \| \| CP-MA \| 0.2 \| -0.1 \| 0.5 \| 0.28 \| \| PO-MA \| 0.2 \| -0.1 \| 0.5 \| 0.39 \| \| SN-MA \| -0 \| -0.3 \| 0.3 \| 1.00 \| \| PO-CP \| -0 \| -0.4 \| 0.3 \| 0.99 \| \| SN-CP \| -0.2 \| -0.6 \| 0.1 \| 0.26 \| \| SN-PO \| -0.2 \| -0.5 \| 0.1 \| 0.37 \| |
| R-codes (abbreviated) provided by the vegan package (Oksanen et al. 2007, see bibliography in the main text)  disJ <- vegdist(Windows16 ,method="jaccard" and “morisita”)  groups <- "MA" "CP","PO","SN"  modJ <- betadisper(disJ, groups)  anova(modJ)  permutest(modJ, pairwise = TRUE, permutations = 999)  mod.HSD <- TukeyHSD(modJ) | | |

Figure S1a&b. Permutation test for homogeneity of multivariate dispersion using Jaccard (a) and Morisita-Horn (b) Compositional Dissimilarity did not reveal significant differences between mountains. MA-La Malinche, CP – Cofre de Perote, PO – Pico de Orizaba, SN-Sierra Negra.

| 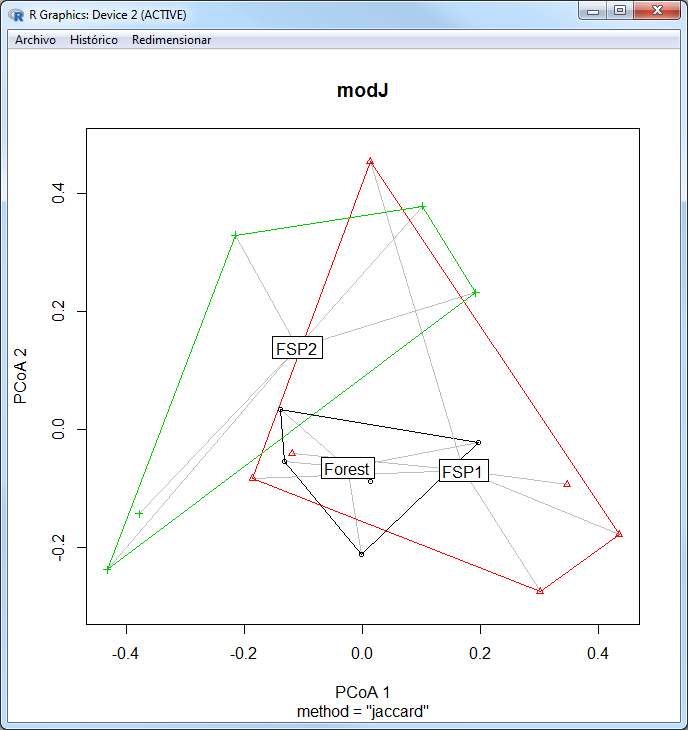  c | Tukey multiple comparisons of means 95% family-wise confidence level  Fit: aov(formula = distances ~ group, data = df)   \| $group \| diff \| lwr \| upr \| p adj \| \| --- \| --- \| --- \| --- \| --- \| \| FSP1-Forest \| 0.06 \| -0.07 \| 0.20 \| 0.43 \| \| FSP2-Forest \| 0.07 \| -0.07 \| 0.21 \| 0.43 \| \| FSP2-FSP1 \| 0.00 \| -0.13 \| 0.14 \| 1.00 \| | |
| --- | --- | --- | --- | --- | --- | --- | --- | --- | --- | --- | --- | --- | --- | --- | --- | --- | --- | --- | --- | --- | --- | --- |
| 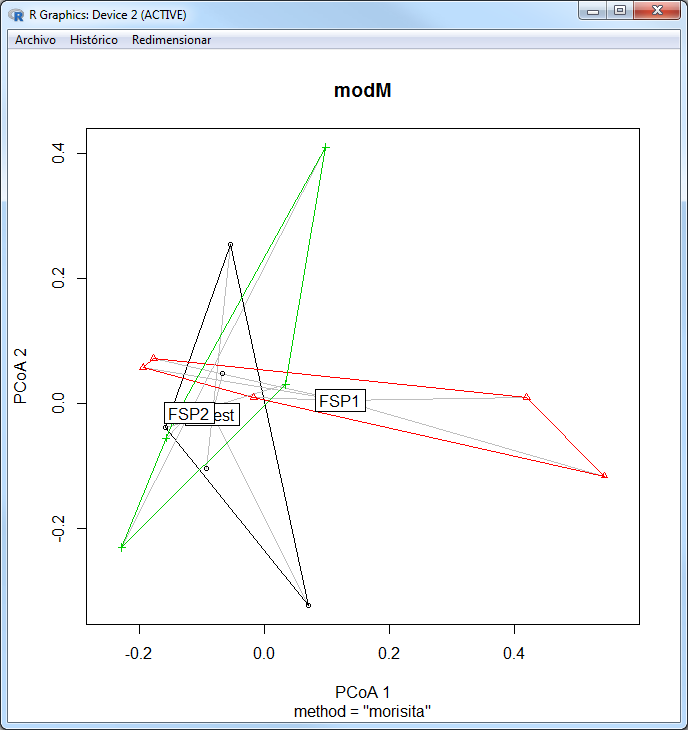  d | | \| $group \| diff \| lwr \| upr \| p adj \| \| --- \| --- \| --- \| --- \| --- \| \| FSP1-Forest \| 0.18 \| -0.09 \| 0.45 \| 0.22 \| \| FSP2-Forest \| 0.16 \| -0.12 \| 0.44 \| 0.33 \| \| FSP2-FSP1 \| -0.02 \| -0.29 \| 0.25 \| 0.98 \| |
| R-codes (abbreviated) provided by the vegan package (Oksanen et al. 2007, see bibliography in the main text)  disJ <- vegdist(Windows16 ,method="jaccard" and “morisita”)  groups <- "Forest" "FSP1","FSP2"  modJ <- betadisper(disJ, groups)  anova(modJ)  permutest(modJ, pairwise = TRUE, permutations = 999)  mod.HSD <- TukeyHSD(modJ) | | |

Figure S1c&d. Permutation test for homogeneity of multivariate dispersion using Jaccard (c) and Morisita-Horn (d) Compositional Dissimilarity did not reveal significant differences between FSP mosaic types. Forest: POWI, MAES, MAWS, CPEI, CPWS; FSP1: CPES, MAEI, MAWI, SNWS, POES; FSP2: POEI, CPWI, SNEI, SNES, SNWI, POWS. Forest – forest dominated sampling unit, FSP1 – Forest had a similar area as scrubland and pasture. FSP2 – forest was less abundant than scrubland and pasture. Classification was based on visual revision at each sampling site.

MA-La Malinche, CP – Cofre de Perote, PO – Pico de Orizaba, SN-Sierra Negra; W- western leeward side, E – eastern windward side, I- inferior elevation (2700 MASL), S – superior (3400 MASL).

a

b

Figure S2 a) Species taxonomic affiliation per mountain; S-Scarabaeinae; A- Aphodiinae; G- Geotrupinae; b) Dispersion patterns according to richness and abundance; NT- Neotropical, NA- Nearctic, MP- Montane Paleoamerican, MM- Meso-American Montane, HP- Mexican High Plateau. Mountains abbreviations: MA - Malinche, CP - Cofre de Perote, PO - Pico de Orizaba, SN - Sierra Negra

Table S1. Number of individuals per species per years at each volcano (sampling months at MA: June 2011, Aug 2012, July 2013; CP: Aug 2011, July 2012, June 2013; PO July 2011, June 2012, Aug 2013; SN: July 2011, June 2012, Aug 2013).

| Species | MA11 | MA12 | MA13 | CP11 | CP12 | CP13 | PO11 | PO12 | PO13 | SN11 | SN12 | SN13 |
| --- | --- | --- | --- | --- | --- | --- | --- | --- | --- | --- | --- | --- |
| *Agrilinellus azteca* | 1 | 0 | 1 | 0 | 17 | 85 | 4 | 46 | 51 | 0 | 0 | 13 |
| *Agrilinellus ornatus* | 0 | 0 | 22 | 4 | 2 | 0 | 1 | 3 | 44 | 0 | 0 | 43 |
| *Blackburneus charmionus* | 0 | 0 | 0 | 0 | 0 | 0 | 0 | 0 | 1 | 0 | 0 | 0 |
| *Blackburneus guatemalensis* | 1 | 0 | 0 | 0 | 0 | 0 | 2 | 32 | 0 | 0 | 0 | 0 |
| *Blackburneus saylorea* | 0 | 0 | 0 | 0 | 0 | 0 | 0 | 0 | 2 | 0 | 0 | 0 |
| *Cephalocyclus hogei* | 0 | 0 | 0 | 0 | 138 | 139 | 4 | 32 | 1 | 0 | 1 | 0 |
| *Ceratotrupes bolivari* | 17 | 12 | 1 | 0 | 4 | 5 | 0 | 1 | 0 | 1 | 0 | 0 |
| *Copris armatus* | 39 | 31 | 28 | 0 | 0 | 0 | 0 | 0 | 0 | 0 | 2 | 2 |
| *Gonaphodiellus bimaculosus* | 0 | 0 | 0 | 0 | 0 | 0 | 4 | 9 | 3 | 0 | 0 | 0 |
| *Gonaphodiellus ophisthius* | 0 | 0 | 13 | 0 | 0 | 2 | 1 | 11 | 90 | 0 | 0 | 0 |
| *Halffterius rufoclavatus* | 0 | 0 | 0 | 0 | 9 | 15 | 0 | 0 | 4 | 0 | 0 | 0 |
| *Labarrus pseudolividus* | 0 | 0 | 0 | 0 | 0 | 0 | 0 | 0 | 0 | 0 | 0 | 1 |
| *Neotrichonotulus inurbanus* | 0 | 0 | 0 | 1 | 0 | 0 | 0 | 0 | 0 | 0 | 0 | 0 |
| *Onthophagus mexicanus* | 2 | 4 | 5 | 0 | 0 | 0 | 0 | 0 | 0 | 0 | 0 | 0 |
| *Onthophagus aureofuscus* | 0 | 0 | 0 | 89 | 25 | 0 | 13 | 0 | 0 | 0 | 0 | 0 |
| *Onthophagus bolivari n.sp.* | 81 | 7 | 84 | 0 | 0 | 0 | 0 | 0 | 0 | 0 | 0 | 0 |
| *Onthophagus ch. chevrolati* | 118 | 90 | 112 | 145 | 250 | 185 | 154 | 179 | 89 | 27 | 115 | 31 |
| *Onthophagus lecontei* | 108 | 7 | 12 | 3 | 4 | 0 | 0 | 0 | 0 | 0 | 0 | 0 |
| *Onthotrupes herbeus* | 12 | 3 | 4 | 4 | 6 | 1 | 226 | 42 | 26 | 1 | 0 | 1 |
| *Onthotrupes nebularum* | 5 | 0 | 0 | 0 | 2 | 0 | 32 | 24 | 50 | 0 | 0 | 0 |
| *Onthotrupes sallei* | 0 | 0 | 0 | 5 | 0 | 0 | 0 | 0 | 0 | 0 | 0 | 0 |
| *Oscarinus indutilis* | 0 | 0 | 0 | 1 | 0 | 0 | 1 | 0 | 0 | 0 | 0 | 0 |
| *Oxyomussetosopunctatus* | 0 | 0 | 0 | 0 | 0 | 0 | 0 | 9 | 7 | 0 | 0 | 0 |
| *Phanaeus amethystinus* | 0 | 0 | 0 | 0 | 0 | 0 | 0 | 0 | 2 | 0 | 0 | 0 |
| *Phanaeus quadridens* | 0 | 0 | 4 | 0 | 0 | 0 | 0 | 0 | 0 | 0 | 0 | 0 |
| *Planolinellus vittatus* | 0 | 0 | 0 | 0 | 0 | 1 | 0 | 0 | 5 | 0 | 0 | 0 |
| *Trichonotuloides alfonsinae n. sp.* | 0 | 0 | 0 | 0 | 0 | 0 | 0 | 6 | 0 | 0 | 2 | 0 |
| *Trichonotuloides feryi n. sp.* | 0 | 0 | 0 | 0 | 1 | 0 | 0 | 0 | 0 | 0 | 0 | 0 |
| *Trichonotuloides glyptus* | 0 | 0 | 0 | 4 | 8 | 0 | 0 | 0 | 0 | 0 | 0 | 0 |
|  |  |  |  |  |  |  |  |  |  |  |  |  |
| Richness per year | 10 | 7 | 11 | 9 | 12 | 8 | 11 | 12 | 14 | 3 | 4 | 6 |
| Abundance | 394 | 161 | 297 | 265 | 478 | 441 | 453 | 406 | 389 | 32 | 124 | 97 |

Table S2. Number of individuals sampled via direct collection inside gopher nests, species per years at the four volcanoes combined.

| SPECIES | Y2011 | Y2012 | Y2013 |
| --- | --- | --- | --- |
| Onthophagus hippopotamus Harold 1869 | 21 | 127 | 74 |
| Geomyphilus barrerai (Islas 1955) | 0 | 67 | 52 |
| Geomyphilus pierai (Lobo & Deloya 1995) | 2 | 89 | 28 |
| Neotrichonotulus perotensis (Deloya & Lobo 1996) | 0 | 102 | 3 |
| **Richness** | **3** | **4** | **4** |
| **Number of individuals** | **23** | **385** | **157** |

Table S3 a and b. Temporal dynamics between pairs of mountains and years. Table a indicates values for Jaccard Similarity Index (^0^CS, mean = 0.3), and the last row indicates species richness at each mountain at each year.Table b shows: lower left values indicate Morisita-Horn compositional similarity (^2^CS) of the whole community (mean =0.62), upper right values show ^2^CS excluding the most abundant species (half of the total individuals) *Onthophagus c. chevrolati* from analysis (mean=0.16).

| a  Jaccard | MA2011 | MA2012 | MA2013 | CP2011 | CP2012 | CP2013 | PO2011 | PO2012 | PO2013 | SN2011 | SN2012 | SN2013 |
| --- | --- | --- | --- | --- | --- | --- | --- | --- | --- | --- | --- | --- |
| MA2011 |  |  |  |  |  |  |  |  |  |  |  |  |
| MA2012 | 0.700 |  |  |  |  |  |  |  |  |  |  |  |
| MA2013 | 0.615 | 0.636 |  |  |  |  |  |  |  |  |  |  |
| CP2011 | 0.188 | 0.231 | 0.250 |  |  |  |  |  |  |  |  |  |
| CP2012 | 0.375 | 0.267 | 0.353 | 0.400 |  |  |  |  |  |  |  |  |
| CP2013 | 0.286 | 0.250 | 0.357 | 0.133 | 0.429 |  |  |  |  |  |  |  |
| PO2011 | 0.313 | 0.125 | 0.294 | 0.333 | 0.438 | 0.357 |  |  |  |  |  |  |
| PO2012 | 0.375 | 0.188 | 0.353 | 0.167 | 0.412 | 0.429 | 0.643 |  |  |  |  |  |
| PO2013 | 0.200 | 0.105 | 0.250 | 0.150 | 0.368 | 0.467 | 0.471 | 0.529 |  |  |  |  |
| SN2011 | 0.300 | 0.429 | 0.273 | 0.200 | 0.250 | 0.375 | 0.167 | 0.250 | 0.133 |  |  |  |
| SN2012 | 0.167 | 0.222 | 0.154 | 0.083 | 0.143 | 0.200 | 0.154 | 0.231 | 0.125 | 0.167 |  |  |
| SN2013 | 0.333 | 0.300 | 0.417 | 0.250 | 0.286 | 0.273 | 0.308 | 0.286 | 0.250 | 0.286 | 0.250 |  |
| Richness | 10 | 7 | 11 | 9 | 12 | 8 | 11 | 12 | 14 | 3 | 4 | 6 |

| b  Morisita | MA2011 | MA2012 | MA2013 | CP2011 | CP2012 | CP2013 | PO2011 | PO2012 | PO2013 | SN2011 | SN2012 | SN2013 |
| --- | --- | --- | --- | --- | --- | --- | --- | --- | --- | --- | --- | --- |
| MA2011 | 1 | *0.558* | *0.699* | *0.027* | *0.029* | *0.008* | *0.082* | *0.058* | *0.034* | *0.139* | *0.182* | *0.015* |
| MA2012 | 0.725 | 1 | *0.485* | *0.010* | *0.019* | *0.011* | *0.079* | *0.045* | *0.017* | *0.293* | *0.587* | *0.039* |
| MA2013 | 0.834 | 0.811 | 1 | *0.015* | *0.010* | *0.008* | *0.041* | *0.052* | *0.191* | *0.037* | *0.199* | *0.230* |
| CP2011 | 0.527 | 0.794 | 0.637 | 1 | *0.177* | *0.000* | *0.101* | *0.019* | *0.021* | *0.031* | *0.000* | *0.044* |
| CP2012 | 0.549 | 0.813 | 0.659 | 0.785 | 1 | *0.894* | *0.072* | *0.409* | *0.071* | *0.050* | *0.322* | *0.049* |
| CP2013 | 0.475 | 0.697 | 0.575 | 0.630 | 0.941 | 1 | *0.030* | *0.542* | *0.212* | *0.026* | *0.282* | *0.149* |
| PO2011 | 0.400 | 0.546 | 0.444 | 0.518 | 0.515 | 0.433 | 1 | *0.443* | *0.230* | *0.694* | *0.006* | *0.031* |
| PO2012 | 0.599 | 0.832 | 0.712 | 0.747 | 0.867 | 0.846 | 0.687 | 1 | *0.559* | *0.309* | *0.161* | *0.168* |
| PO2013 | 0.385 | 0.498 | 0.533 | 0.448 | 0.492 | 0.528 | 0.465 | 0.703 | 1 | *0.131* | *0.003* | *0.397* |
| SN2011 | 0.525 | 0.867 | 0.647 | 0.805 | 0.800 | 0.667 | 0.544 | 0.762 | 0.430 | 1 | *0.000* | *0.016* |
| SN2012 | 0.515 | 0.859 | 0.640 | 0.797 | 0.795 | 0.662 | 0.511 | 0.746 | 0.418 | 0.998 | 1 | *0.029* |
| SN2013 | 0.364 | 0.541 | 0.557 | 0.499 | 0.513 | 0.506 | 0.338 | 0.577 | 0.590 | 0.516 | 0.511 | 1 |
